# Retinal image mosaicking using scale-invariant feature transformation feature descriptors and Voronoi diagram (Erratum)

**DOI:** 10.1117/1.JMI.7.4.049801

**Published:** 2020-07-28

**Authors:** Jalil Jalili, Sedigheh M. Hejazi, Mohammad Riazi-Esfahani, Arash Eliasi, Mohsen Ebrahimi, Mojtaba Seydi, Masoud Aghsaei Fard, Alireza Ahmadian

**Affiliations:** a Tehran University of Medical Sciences, School of Medicine, Medical Physics and Biomedical Engineering Department, Tehran, Iran; b Tehran University of Medical Sciences, Imam Khomeini Hospital, Advanced Medical Technologies and Equipment Institute Research Center for Molecular and Cellular in Imaging, Bio-Optical Imaging Group, Tehran, Iran; c University of California Irvine, Gavin Herbert Eye Institute, Department of Ophthalmology, Irvine, California, United States; d Tehran University of Medical Science, Farabi Eye Hospital BB, Eye Research Center, Tehran, Iran

## Abstract

The erratum corrects an error in the originally published article.

This article [*J. Med. Imag.* **7**(4), 044001 (2020) doi: 10.1117/1.JMI.7.4.044001] was originally published online on 15 July 2020 with an error in the surname of the 5^th^ author. Previously, the surname was listed as Fard; the correct surname is Aghsaei Fard.

This error was corrected on 20 July 2020.

